# Colpocleisis—Still a Valuable Option: A Point of Technique

**DOI:** 10.3390/jcm14207433

**Published:** 2025-10-21

**Authors:** Diana Hoehn, Hannes Egli, Martin Chase Marak, Gloria Ryu, Anna-Sophie Villiger, Giovanni Ruggeri, Michael David Mueller, Annette Kuhn

**Affiliations:** 1 Division of Urogynaecology, Department of Obstetrics and Gynaecology, Bern University Hospital, University of Bern, 3010 Bern, Switzerland; 2 Department of Statistics, Texas A&M University, College Station, TX 77843, USA

**Keywords:** Le-Fort Colpocleisis, total colpocleisis, purse-string sutures, pelvic organ prolapse, vaginal surgery, obliterative surgery, voiding disorders, recurrence, incontinence, complications

## Abstract

**Background/Objectives**: Pelvic organ prolapse (POP) is a common condition that increases with age and affects up to 40% of women. Colpocleisis is a possible native-tissue repair used in elderly persons not interested in vaginally penetrative sex to correct advanced POP. This study aims to evaluate the recurrence and reoperation rate of a technique using purse-string sutures in a standardised way. **Methods**: This retrospective quality control study evaluated all women who underwent obliterative procedures for POP at the Department of Obstetrics and Gynaecology at the University Hospital of Bern from 2014 to 2023. Total Colpocleisis (TC) and Le-Fort Colpocleisis (LFC) were performed by a standardised technical procedure using purse-string sutures. The primary outcome was the recurrence rate measured by the POP-Q stage (stage 2 or higher). Reoperation rate, perioperative complications, bladder outlet disorders and incontinence symptoms were assessed as secondary outcomes. **Results**: We analysed eighty-eight patients who underwent obliterative surgery with TC or LFC in this study. The recurrence rate for all patients was 16%, and the reoperation rate was 9.2%. In patients without previous surgeries (52%), the recurrence rate was 7%. Thirteen patients (14.8%) had perioperative complications, mainly urinary tract infections (seven patients, 8%). Objective POP improved significantly (*p* < 0.001), as did the bladder voiding dysfunction in the rate of high postvoid residual volume (*p* < 0.05), stress urinary incontinence, overactive bladder and mixed urinary incontinence (*p* < 0.001). In three patients, de novo stress urinary incontinence developed postoperatively. **Conclusions**: Colpocleisis by the purse-string technique is an effective surgical treatment for advanced POP surgery. Recurrence and reoperation rates are similar to the previously mentioned techniques and are easy to learn due to the standardised procedure.

## 1. Introduction

Pelvic organ prolapse (POP) is a common issue that increases with age, affecting up to 40% of women [1]. Childbirth, obesity, ageing and connective tissue or muscle atrophy can weaken the pelvic floor, leading to the descent of pelvic organs [2]. The most bothersome symptoms of POP for patients are a feeling of bulge, lower abdominal pain, and dyspareunia, which generally occur when the descent trespasses the POP stage 2 [3,4]. In the case of advanced prolapse, other organs, such as the bladder, may also be affected. Bladder emptying disorders can occur from subvesical obstruction, or kinked ureters, causing urinary retention or hydronephrosis. The risk of urinary tract infections and subsequently morbidity rises. In persistent bothersome symptoms, pain or impaired organ function, conservative or operative treatment is necessary. First-line treatment involves the use of pessaries and pelvic floor muscle training [5]. However, pain or dislocation of the pessary can occur in consequence to several reasons. A voluminous body due to obesity, or a lack of visual and manual ability in the ageing population, can limit the handling of the pessary and its correct placement [6]. Surgery will be necessary because of subsequent treatment failure. The lifetime risk of undergoing surgery for POP is up to 12.6% [7]. The medical condition of the patient, restoration of anatomy to accommodate sexual intercourse and site-specific defects of the endopelvic fascia are factors influencing the choice to restore anatomy or gain function of the pelvic support.

In the past, obliterative procedures were considered ethically unacceptable and were therefore abandoned as a surgical option. Following the ban or broad restriction on transvaginal meshes in 2019, reconstructive or obliterative vaginal surgeries with native tissue repair have become increasingly interesting [8,9,10].

However, with an ageing, obese and multimorbid society, minimally invasive vaginal procedures are becoming important again. Although the technique described in the available literature is standardised and produces excellent results in terms of recurrence rates, there is potential to further optimise the technique in terms of operating time and recurrence rate.

The Le-Fort Colpocleisis obliterative procedure can be an appropriate choice to treat advanced POP in elderly, polymorbid and non-vaginally sexually active persons without the need of hysterectomy. The technique was described in 1877 by Leon Le-Fort and in 1881 by Neugebauer with the same scope and is one of the first described gynaecologic surgeries for POP [10,11]. This technique is indicated for women with posthysterectomy vaginal vault prolapse and also for women with advanced uterovaginal prolapse. The original description of the procedure involves the denudation of the vaginal skin anteriorly and posteriorly in a strip-shaped manner. Interrupted sutures approximate and plicate the anterior and posterior vaginal walls. Back then, the sutures intended to stabilise the relaxation of the vaginal skin as described by Le-Fort. Over time, the technique has been improved to minimise recurrence rates by a wider denudation of the vaginal skin and narrowing the lateral channels to ensure drainage of secretions from the vagina and uterus. A perineorraphy can be performed to reduce the recurrence rate in cases of extended genital hiatus.

Since the procedure’s first description, various suture materials have been used. Until now, it is unclear whether non-absorbable sutures should be preferred to minimise the recurrence rate.

In our clinic, we exclusively performed the modified purse-string suture for both LFC and TC. As with interrupted sutures, they achieve the inversion of the vagina with the advantage to reduce the amount of suture material and therefore the operative time.

The objective of this retrospective study was to assess the recurrence and the reoperation rate for obliterative procedures adopting a purse-string suture approach.

## 2. Materials and Methods

This is a single-centre, retrospective, quality control study at a tertiary referral centre at the Department of Obstetrics and Gynaecology of the University Hospital of Bern, Switzerland. All patients underwent obliterative surgical procedures by Le-Fort Colpocleisis (LFC) and Total Colpocleisis (TC) using the purse-string technique between January 2013 to December 2023 and were analysed by follow-up until December 2024.

Inclusion criteria were age > 18 years old, signed informed consent, severe pelvic organ prolapse according to the ICS/IUGS POP-Q system, willingness to undergo an obliterative procedure with the sexual consequences, follow-up at least 6 weeks.

A Pelvic Organ Prolapse-Quantification system was assessed [4] as indicated by the standardised examination of the ICS/IUGA POP-Q classification system by a trained urogynaecologist in lithotomy position with maximal straining.

We evaluated LFC and TC by the modified technique.

### 2.1. Surgical Procedure of the Modified LFC with Purse-String Sutures

**Figure 1:**
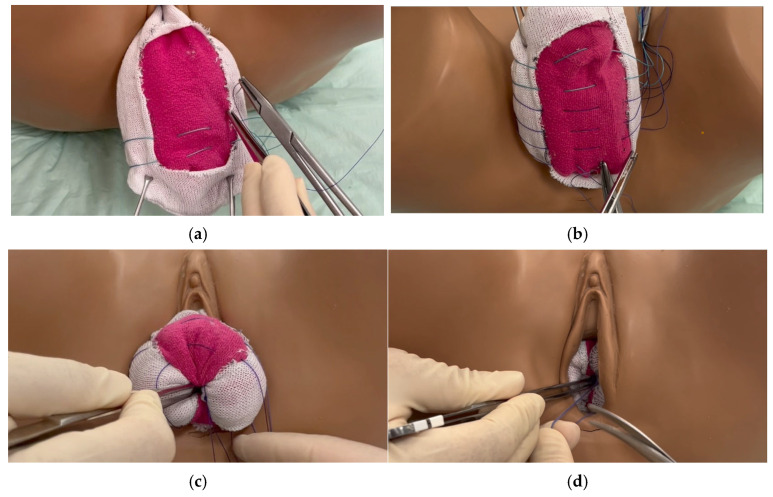
(**a**) Stitches on the anterior vaginal wall in a purse-string manner, running anteriorly to posteriorly. (**b**) Six stitches are set. Two polyester threads 2-0 (green) apically, three polydioxanone 2-0 and one distally with polyglactin 2-0. (**c**) Inversion of the vagina using a blunt tip and knotting the first apical suture, followed by the next apical suture. (**d**) Complete inversion of the vagina after knotting the last suture.

(1) Placement of a 16-gauge indwelling catheter.
(2) Two identical vertical rectangles are marked anteriorly and posteriorly, closely from the apex to the vaginal introitus, but not further than 3 cm from the meatus urethrae externus. These rectangles should be approximately 3 cm apart, which corresponds to the urethrovesical junction. The suburethral part is not excised in anticipation of the need for a future tension-free vaginal tape in case of stress urinary incontinence.
(3) The vagina is infiltrated with saline or a local anaesthetic. This step helps to separate the epithelium from the endopelvic fascia and optimise dissection.
(4) Denudation of the rectangles. Only the epithelium should be excised in order to conserve as much of the endopelvic fascia as possible. Careful haemostasis is applied using bipolar forceps and tampons.
(5) Close the vagina using purse-string sutures running from the right border to the left border of the anterior part of the rectangle, and then from the left border to the right border of the posterior part of the rectangle (Figure 1a,b). The first suture, made of non-absorbable thread, Ethibond^®^ Excel 2-0 (Polyester; Ethicon, LLC., Somerset County, NJ, USA), is placed at the distal end of the cervix. Depending on the length of the vagina, another suture can be placed 1 cm apart to ensure stability. In extensive urogenital atrophy with thin endopelvic fascia, absorbable sutures are used to avoid perforation.
(6) Further sutures with PDS™ II 2-0 (polydioxanone; 2-0 Ethicon, Somerset County, NJ, USA) are placed in the same manner, approximately 1 cm apart. The sutures must be placed deep in the tissue to ensure stability. The position of the sutures should not be too close together to avoid difficulty in knotting, nor should they be too far apart for stability reasons. Distally, fast-resorbable sutures Vicryl^®^ 2-0 (Polyglactin 910; Ethicon, Somerset County, NJ, USA) are used. A total of 5–10 sutures will be placed according to the length of the vagina.
(7) The most apical, and therefore distal, suture will be knotted first. The vagina will be inverted by applying pressure to the tissue with a blunt instrument (Figure 1c) and bringing the anterior and posterior parts together. All sutures will be knotted from distally to proximally. At the end, the vagina will be completely inverted (Figure 1d).

According to the anatomy and the surgeons’ preference, a complementary colpoperineoplasty will be applied to improve the surgical outcome by increasing the stability of the procedure.

In TC, the whole vaginal epithelium is divided into four quadrants and will be denuded up to 3 cm from the vaginal hymen. The same type of stitching is applied.

Patients who had undergone a hysterectomy either prior to or during surgery received TC. Those who had not undergone a hysterectomy received LFC. However, LFC was also given to women if TC was not technically feasible—for example, in cases of a short and wide vagina, a significant prolapse with a very long and wide vagina or a recurrence after TC.

The primary outcome measure was the POP-Q stage, as determined by the Pelvic Organ Prolapse-Quantification system, to assess recurrence. A POP-Q stage ≥ 2 was defined as a recurrence. Secondary outcome measures were the reoperation rate, time of surgery, blood loss, perioperative complications (Clavien–Dindo) [12], high residual bladder volume defined as >100 mL [13], incontinence symptoms accordingly to the ICS terminology [14] and mode of anaesthesia.

Ethical approval for this study was obtained from the Ethics Commission of the Canton of Bern, Switzerland (reference number: KEK 2024-01304). The study was conducted following the guidelines outlined in the Declaration of Helsinki (DoH), the Essentials of Good Epidemiological Practice issued by Public Health Switzerland (EGEP) and the standards set forth by Swiss Law. This study adheres to the reporting standards outlined in the Strengthening the Reporting of Observational Studies in Epidemiology (STROBE) statement.

### 2.2. Statistical Analysis

All analyses were performed post hoc from data collected following the surgical procedures. A *t*-test was used to compare differences in surgery time. For continuous outcomes, a linear regression model adjusted for baseline was used. Binary outcomes were fitted using a logistic regression adjusted for baseline or study days, as appropriate. A McNemar test was performed for incontinence and urine outcomes to determine if the procedure had a significant effect on these symptoms. POP-Q scores were summarised on both a continuous and a discrete scale. A within-participant analysis was conducted for participants who measured a POP-Q score at all three time points—baseline, 6 weeks post-surgery and the last follow-up. An interaction between procedure type and previous surgery was added to assess potential covariation between these variables. A Kaplan–Meier survival curve was fitted to assess time to recurrence for all participants. A participant was censored at their last follow-up date if no recurrence had occurred. A Cox proportional hazards model was fit to assess the hazard ratio between groups. Due to the post hoc, the exploratory nature of this analysis and with a limited sample size, no multiplicity adjustment was performed in order to conserve power. All data analyses were conducted using R software (version 4.5.0).

## 3. Results

Eighty-eight patients underwent obliterative procedures during the study period. Of those, 49 patients underwent LFC and 39 underwent TC. The baseline characteristics of the POP-Q score in Table 1 shows no difference between groups. The patients’ characteristics and the perioperative assessment, including the surgical procedures, are reported in Table 1. In fifteen patients (38.5% of TC), a concomitant vaginal hysterectomy with TC was performed. Additional surgery included vaginal hysterectomy (*n* = 15), laparoscopic hysterectomy (*n* = 1), haemorrhoidectomy (*n* = 1), haemorrhoidopexie (*n* = 1), posterior colporrhaphy (*n* = 5), perineorrhaphy (*n* = 2), anterior colporrhaphy (*n* = 4), retropubic mid-urethral sling (*n* = 2), hysteroscopy (*n* = 2), cystofix (*n* = 5) and posterior MESH insertion (*n* = 1). Most patients who underwent LFC (33/49) were undergoing their first POP procedure. Forty-two women had previously undergone surgery, including vaginal hysterectomy (*n* = 19), laparoscopic hysterectomy (*n* = 1), abdominal supracervical hysterectomy (*n* = 1), abdominal hysterectomy (*n* = 20), sacrospinous fixation (*n* = 10), posterior colporrhaphy (*n* = 5), anterior colporrhaphy (*n* = 6), uteropexy (*n* = 1), laparoscopic sacrocolpopexy (*n*= 1), laparoscopic rectopexy (*n* = 2), colpocleisis (*n* = 1) and unknown (*n* = 4). The LFC surgery time was significantly shorter (*p* = 0.01) than the TC procedure by 23 min.

Table 2 displays the results of baseline and 6-week post-surgery POP-Q scores for all participants.

**Figure 2:**
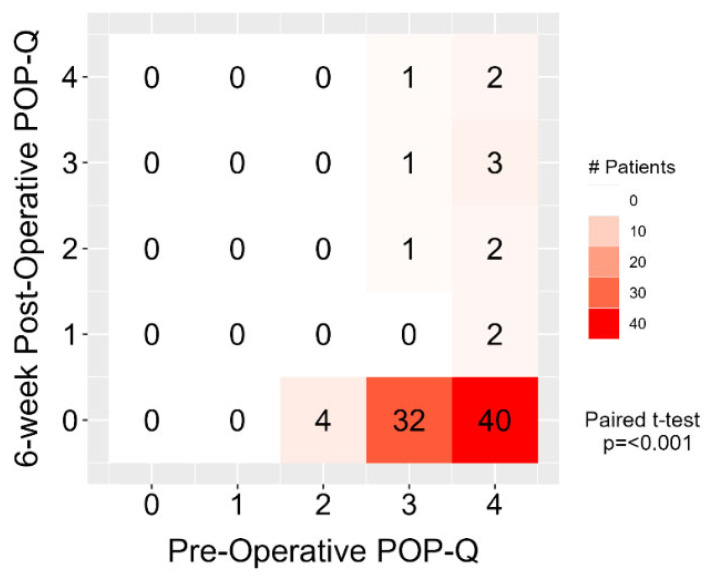
Heatmap of POP-Q stage during baseline and 6 weeks post-surgery. Numbers inside each cell represent number of patients. Forty patients with POP-Q stage 4 and thirty-two with POP-Q stage 3 had no recurrence after 6 weeks. Two patients with POP-Q stage 4 baseline had a POP-Q stage 4 after 6 weeks.

Figure 2 displays the results of baseline and 6-week post-surgery POP-Q scores for all participants.

The odds of prolapse recurrence in patients who have had a previous prolapse procedure were 5 times higher (Table 3) than those who are receiving the procedure for the first time (*p* = 0.02). A patient with diabetes had 4 times greater odds of recurrence than those with no diabetes (*p* = 0.04).

The procedure was shown to have a significant effect on both incontinence and residual urine for the cohort (*p* < 0.001 for both). Although all incontinence groups showed at least a 40% improvement, those with mixed incontinence showed the most significant improvement at 85% (Table 4).

**Figure 3:**
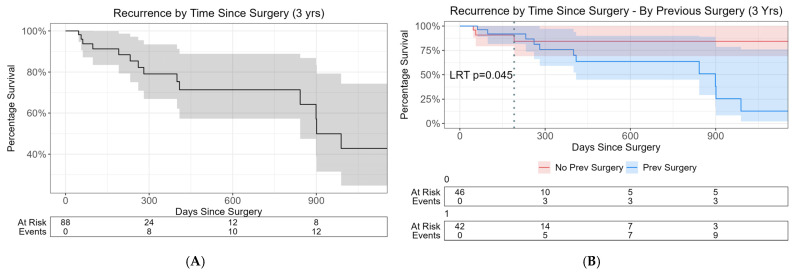
Kaplan–Meier survival curves of rate of prolapse recurrence. (**A**) All participants across the entire follow-up time; grey area demonstrating the 95% CI and the black line the 1 - recurrence probability (**B**) Cox PH model for previous prolapse surgery vs. no previous prolapse surgery at approximately 3 years (HR = 3.4, *p* = 0.045).

The Kaplan–Meier survival curve [Figure 3] showed that the probability of no recurrence at 1 year was 80%. At 2 years, the probability of no recurrence was 70%. Additionally, patients with a previous surgery had a 3.4 times higher probability of recurrence than those without a previous surgery, at any time point (*p* = 0.045).

The last day of recurrence for those without a previous surgery was 191 days (Figure 3B, dotted line), compared to 989 days in the previous surgery group.

Nine of the 88 patients experienced a recurrence after LFC/TC, requiring reoperation: One received an LFC; five patients received a TC; two received a sacrocolpopexy; and one received a vaginal mesh implant.

Table 5 shows the results of the perioperative complications according to the Clavien–Dindo Classification.

## 4. Discussion

The current study confirms that modified LFC and TC are safe and effective procedures in an elderly and morbid population.

Surgery under local anaesthesia is preferable for older people because of concerns about cognitive function. However, this is not always possible due to the length of the operation, which depends on factors such as the extent of the prolapse. TC, for example, takes an average of 23 min (*p* > 0.01) longer, as does surgery on previously operated patients.

Aligning with previously published data, the rate of postoperative complications was low, with most events classified as Clavien Dindo grade I [15]. Urinary tract infections were the most common complications. These were treated with antibiotics according to the antibiogram.

One postoperative wound infection did occur, and two patients received patient blood management in consequence to intensive blood loss. One postoperative haemorrhage needing surgery was detected. Overall, blood loss was marginal. Minimal blood loss was quantified at 50 mL and ranged from 50 to 700 mL (mean 110.8 mL, median 50 mL).

A severe descent of the pelvic organs can cause infravesical bladder outlet obstruction with significant PVR and succeeding morbidity.

One patient suffered from urosepsis before surgery due to voiding dysfunction caused by prolapse. Forty-five per cent (*n* = 39) of patients had high PVR before surgery and needed an indwelling catheter or intermittent self-catheterisation. Postoperatively, PVR improved significantly (*p* < 0.001). Nevertheless, 18% of patients reported still augmented PVR. One reason can be the decay of muscle tissue and transformation to connective tissue in elderly patients by hypoperfusion and atrophy [2]. Another reason is that POP leads to an elevated residual volume as a result of the chronic overdistension of the detrusor muscle [16]. In this case, PVR is assumed to improve over time, but not if tissue transformation occurred.

In our study, patients who underwent previous pelvic surgery have an OR of 2.93 for high residual urine after LFC or TC. As previous research shows, postoperative bladder dysfunction can be a common condition after gynaecologic surgery [17,18].

Stress incontinence, MUI and OAB generally improved after the operation. Encouragingly, more women reported an improvement in incontinence than a de novo stress or urge incontinence. Usually, a higher rate for de novo stress urinary incontinence is observed because of anatomy restoration and low urethral pressure.

The correlation between OAB and prolapse has not been well defined. However, a study investigating risk factors for OAB after surgical prolapse repair [19] demonstrated that preoperative OAB was associated with persistence of OAB after POP surgery, as were postoperative SUI and voiding symptoms. Moreover, they found a correlation in only 36% between OAB and detrusor overactivity in urodynamics. In our study, we could not support this finding. OAB symptoms were relieved, and just three patients had persistent OAB. Two patients reported SUI relief but developed de novo OAB.

To the best of our knowledge, this is the first study to compare LFC to TC. POP improved significantly after surgery in LFC (*p* < 0.001) and TC (*p* < 0.001). LFC performed slightly better than TC, with a score of −0.27 in the POP-Q, but without a clinically significant difference (*p* = 0.21). TC has an odds ratio of 2.4, which is likely related to the higher number of previous surgeries, as the surgical outcome was generally worse for patients who had previously undergone surgery (POP −0.48, *p* = 0.036). Prior pelvic surgery is therefore an important predictor for recurrence. Therefore, both procedures are comparable.

Another risk factor related to recurrence is prior pelvic surgery. Scar tissue and altered pelvic anatomy with reduced blood flow can lead to wound-healing disorders and compromise long-term surgical success. Tissue hypoperfusion and hypoxia result as a consequence due to their endothelial dysfunction and structural changes in large and small arteries. These alterations accelerate the process of arterial remodelling and associated senescence that occurs with ageing [20].

The vagina is supplied with blood via several arteries. The upper part of the vagina is mainly supplied by the vaginal branch of the uterine artery. The middle part is supplied by the vaginal artery, which originates from the internal iliac artery or, occasionally, as a branch of the uterine artery. The ligation of the uterine artery during a hysterectomy has been shown to result in reduced blood flow to the upper and middle parts of the vagina. With regard to the endopelvic fascia, this results in a weakening of the sacrouterine and cardinal ligaments, which stabilise the apex (the middle compartment, DeLancey II). Consequently, it is unsurprising that prior surgical interventions, notably hysterectomy, have been demonstrated to heighten the risk of prolapse and suboptimal outcomes, a consequence of diminished perfusion subsequent to uterine artery ligation.

Overall, LFC and TC together had a surgery time of a mean 93.93 min (SD 38.32). LFC took 23 min less than TC (*p* < 0.01). As the entire vaginal skin must be removed during TC, the complete denudation of the vaginal epithelium and haemostasis explain the statistical and clinical difference. In patients with a large vaginal cuff prolapse where extensive vaginal tissue excision is necessary, LFC can be performed instead of TC in cases of polymorbidity and to reduce operating time with equally good results. Compared to our study, reported operating times are longer [21]. For patients who receive a LFC or TC under local anaesthesia or for patients for whom the operation time should be kept as short as possible, our method is certainly preferable to the standard technique due to shorter operation time. In addition to the shorter operation time, the modified technique has the further advantage of reduced suture material consumption, which contributes to the cost-effectiveness of the operation.

Overall recurrence rate was 16%. Persons who underwent pelvic surgery previously had a higher recurrence rate of 27.5%. In patients without previous surgery, the recurrence rate was as low as 7%, according to previous studies, which reported reoperation rates of 6.5% after 2 years and 8.2% after 10 years, with low perioperative risks [22]. Five of nine patients with previous surgery had more than three prolapse procedures in the past, which can explain the high recurrence rate in those patients.

Patients with diabetes mellitus have a higher risk for recurrences with an OR of 4.01. This may be attributed to impaired wound healing and tissue remodelling caused by advanced glycation products (AGEs). In hyperglycaemic conditions, AGE accumulate in connective tissue and induce an abnormal collagen cross-linking. This causes the tissue to become stiff and limits its capacity for remodelling. They also promote oxidative stress and persistent inflammation through activation of the AGE–RAGE (Receptor for Advanced Glycation End-products) axis, which further compromises wound healing and tissue integration [23,24]. These mechanisms provide a plausible biological explanation for how diabetes-related AGE accumulation can weaken the pelvic support structures, thereby increasing the risk of prolapse recurrence following surgery.

In patients with poor tissue properties, absorbable threads were used in order to avoid intra- and postoperative complications. The recurrence rate was 16% and 15%, respectively (*p* = 0.89). The findings show that the choice of suture material does not influence the recurrence rate. Our data correlate with a recently published meta-analysis [25], which in accordance with our study, found no significant difference between absorbable and non-absorbable sutures in patients who underwent native tissue repair (McCall suture, sacrospinal fixation or sacrocolpopexy).

A general limitation of this study is its retrospective character leading to the usual loss to follow-up, varying standards for follow-up and potential bias due to its monocentric study character. There is a potential bias of selection in the assignment of LFC or TC. However, we cannot change this bias as the choice of surgery is selected or changed intraoperatively due to local circumstances. We have shown a five-fold increased risk for recurrence in previously operated patients. However, we must take into consideration that previously operated patients do present a challenging situation, in general, which may add to a bias. The rather short follow-up period is a weakness of the study, and the recurrence rate is possibly underrated. However, in our clinical experience, recurrences do occur early and not after years. Our main intention is to present a novel technique. Longer follow-up of this patient group will follow. In a future prospective setting, follow-up, including validated questionnaires confirming patient reported outcomes, will be performed.

Prospective studies should be conducted to determine the most suitable surgical therapy (standard or modified) for elderly patients with comorbidities, with an assessment of cognitive, lower urinary tract symptoms and symptom-based functions.

## 5. Conclusions

The modified LFC and TC techniques are effective and safe surgical treatments for severe POP with low perioperative risks and low serious postoperative complications. A notably higher risk of recurrence is found in patients with a history of prior pelvic surgery and those with diabetes mellitus. We noted no difference in terms of recurrences for either LFC or TC about the types of sutures. Subsequent studies should concentrate more on the localisation of the recurrence site. Further prospective studies are needed to compare standard and modified techniques.

## Figures and Tables

**Table 1:** Baseline patients’ characteristics and surgery summary.

	Overall *n* = 88	LFC *n* = 49	TC *n* = 39
Patient Characteristics			
Age at Surgery (years)			
* Mean ± SD*	82 ± 7	84 ± 7	80 ± 6.5
Age > 80 *n* (%)	56 (64%)	36 (73%)	20 (51%)
BMI (kg/m^2^) Mean ± SD	24.6 ± 4.8	24.7 ± 4.9	24.4 ± 4.7
Diabetes—Yes *n* (%)	14 (16%)	8 (16%)	6 (15%)
Number of Deliveries ^a^ Mean ± SD	2.6 ± 1.6	2.6 ± 1.1	2.6 ± 2.0
Smoker—Yes ^a^ *n* (%)	4 (5%)	3 (6%)	1 (3%)
Previous Prolapse Surgery—Yes *n* (%)	42 (48%)	16 (33%)	26 (67%)
Additional Surgery—Yes *n* (%)	37 (42%)	19 (39%)	18 (46%)
Residual Urine—Yes—*n* (%) ^a^	39 (45%)	22 (47%)	17 (44%)
Catheter—Yes *n* (%) ^a^	16 (18%)	9 (19%)	7 (18%)
Incontinence—*n* (%)			
None	45 (51%)	24 (50%)	20 (51%)
Stress	14 (16%)	9 (19%)	5 (13%)
Overactive	9 (10%)	5 (10%)	4 (10%)
Mixed	20 (23%)	10 (21%)	10 (26%)
POP-Q Score Baseline			
Mean ± SD	3.5 ± 0.6	3.5 ± 0.6	3.5 ± 0.6
0—*n* (%)	0 (0%)	0 (0%)	0 (0%)
1—*n* (%)	0 (0%)	0 (0%)	0 (0%)
2—*n* (%)	4 (5%)	2 (4%)	2 (5%)
3—*n* (%)	35 (40%)	20 (41%)	15 (38%)
4—*n* (%)	49 (56%)	27 (55%)	22 (56%)
Surgery Summary			
Surgery Time (mins) ^a^ Mean ± SD	94 ± 38	84 ± 34	107 ± 41
Anaesthesia Type *n* (%)			
Spinal	27 (31%)	19 (39%)	8 (21%)
General	56 (64%)	27 (55%)	29 (74%)
Peridural	2 (2%)	1 (2%)	1 (3%)
Local	3 (3%)	2 (4%)	1 (3%)
Thread Type n (%) *^a^			
Absorbable and Non-Absorbable	62 (77%)	27 (63%)	35 (92%)
Absorbable Only	19 (23%)	16 (37%)	3 (8%)
Additional Surgery—Yes *n (%)*	37 (42%)	19 (39%)	18 (46%)

* Difference between LeFort and Total surgery minutes (*t*-test): −23 (−38, −7) [0.005]. ^a^—Deliveries unknown for 8, smoking status unknown for 4, Thread type unknown for 7, catheter unknown for 1, and residual unknown for 2 participants.

**Table 2:** Anatomical and functional outcomes at 6 weeks follow-up.

Metric	Baseline		6 Weeks Post-Surgery		Comparison
POP-Q Stage (6 weeks)	*n*	Mean ± SD	*n*	Mean ± SD	Adj. Difference [*p*-value]
**All Patients**	88	3.5 ± 0.6	88	0.4 ± 1.0	−3.2 (−3.4, −3.0) [<0.001] ^a^
Le-Fort Colpocleisis	49	3.5 ± 0.6	49	0.2 ± 0.8	0.3 (−0.2, 0.7) [0.21] ^b^
Total Colpocleisis	39	3.5 ± 0.6	39	0.5 ± 1.2	
No Previous Prolapse Surgery	46	3.6 ± 0.5	46	0.2 ± 0.6	0.5 (0.1, 0.9) [0.02] ^b^
Previous Prolapse Surgery	42	3.4 ± 0.6	42	0.6 ± 1.3	
Absorbable Threads Only	19	3.5 ± 0.6	19	0.4 ± 1.0	0.0 (−0.5, 0.5) [0.9] ^b^
Non-Absorbable and Absorbable Threads	62	3.5 ± 0.6	62	0.3 ± 0.9	
**Patients with No Previous Prolapse Surgery ^c^**					
Le-Fort Colpocleisis	33	3.6 ± 0.5	33	0.2 ± 0.7	−0.2 (−0.6, 0.2) [0.24] ^b^
Total Colpocleisis	13	3.7 ± 0.6	13	0 ± 0	
**Patients with Previous Prolapse Surgery ^c^**					
Le-Fort Colpocleisis	16	3.3 ± 0.7	16	0.3 ± 1	0.4 (−0.4, 1.2) [0.30] ^b^
Total Colpocleisis	26	3.4 ± 0.6	26	0.8 ± 1.4	
**Residual Urine—Yes *n (%)***	*n* = 86		*n* = 78		Adj. Difference [*p*-value]
All Patients	39 (45%)		14 (18%)		[<0.001] ^d^
Le-Fort Colpocleisis	22 (47%)		5 (12%)		3.1 (0.8, 12.3) [0.10] ^e^
Total Colpocleisis	17 (44%)		9 (25%)		
No Previous Prolapse Surgery	18 (40%)		4 (11%)		2.7 (0.8, 11.6) [0.14] ^e^
Previous Prolapse Surgery	21 (51%)		10 (24%)		
**Incontinence *n (%)***	*n* = 88		*n* = 80		[*p*-value]
No Incontinence	45 (51%)		61 (76%)		[<0.001] ^d^
Incontinence	43 (49%)		19 (24%)		

^a^—Paired *t*-test. ^b^—Linear regression model adjusted for baseline values. ^c^—Interaction between previous surgery and procedure on POP-Q shown not significant (*p*-value = 0.14). ^d^—McNemar tests to assess procedure effect (Incontinence assessed as improvement from baseline). ^e^—Logistic regression model adjusted to baseline values.

**Table 3:** POP-Q stage in women with a final follow-up visit and recurrence rate summary in the whole study population.

Metric	Summary		Comparison
POP-Q Stage (Last Follow-Up) ^a^	*n*	Mean ± SD	Adj. Difference [*p*-value] ^b^
Baseline	51	3.6 ± 0.5	−3.0 (−3.3, −2.7) [<0.001] (Follow-up to Baseline)
6-Week Post Surgery	51	0.6 ± 1.2	
Last Follow-up Visit (days) Median, IQR (73, 452)	51	0.6 ± 1.2	
Recurrence Rate—%	*n*	*n* (%)	Adj. Difference [*p*-value] ^c^
All patients	88	14 (16%)	-
Le-Fort Colpocleisis	49	5 (10%)	2.4 (0.8, 8.7) [0.15]
Total Colpocleisis	39	9 (23%)	
No Previous Prolapse Surgery	46	3 (7%)	5.4 (1.5, 26.8) [0.02]
Previous Prolapse Surgery	42	11 (26%)	
Absorbable Threads Only	19	3 (16%)	0.9 (0.2, 4.5) [0.89]
Non-Absorbable and Absorbable Threads	62	9 (15%)	
No Diabetes	74	9 (12%)	4.0 (1.0, 14.8) [0.04]
Diabetes	14	5 (36%)	
Age ≤ 80	32	5 (16%)	1.2 (0.4, 4.6) [0.76]
Age > 80	56	9 (16%)	
Deliveries ≤ 2	39	8 (21%)	0.8 (0.2, 2.5) [0.65]
Deliveries > 2	41	5 (12%)	
Non-Smoker	80	14 (18%)	^- d^
Smoker	4	0 (0%)	
BMI < 25	52	9 (17%)	0.6 (0.2, 2.0) [0.40]
BMI ≥ 25	36	5 (14%)	

^a^—Only participants with a final-follow up visit (in addition to the 6-week visit). ^b^—Linear regression model adjusted for baseline values. ^c^—Logistic regression model adjusted for study days. ^d^—No statistical test due to low sample size.

**Table 4:** Incontinence summary.

Incontinence at Baseline	Improving After Surgery *n* (%)	Worsening After Surgery *n* (%)	No Change *n* (%)	No Follow-Up Test *n* (%)
No Incontinence (*n* = 45)	-	3 (7%)	40 (89%)	2 (4%)
Stress Incontinence (*n*= 14)	6 (43%)	1 (7%)	4 (29%)	3 (21%)
Urge Incontinence (*n* = 9)	4 (44%)	1 (11%)	3 (33%)	1 (11%)
Mixed Incontinence (*n* = 20)	17 (85%)	-	1 (5%)	2 (10%)

**Table 5:** The Clavien–Dindo Classification of perioperative and postoperative complications.

	LFC	TC	Treatment
	Patients (*n* = 5)	Patients (*n* = 8)	
Grade I			
- Angina pectoris	0	1	surveillance
Grade II			
- Urinary tract infections	3	4	antibiotics
- Wound infection	0	1	antibiotics
- Anaemia	1	1	transfusion
Grade III			
Grade IIIa			
- urinary retention	1	0	indwelling catheter
Grade IIIb			
- postoperative bleeding	0	1	revision

## Data Availability

The raw data supporting the conclusion of this article will be made available by the corresponding author on request because our data are confidential.
